# Low-Intensity Electrical Stimulation to Improve the Neurological Aspect of Weakness in Individuals with Chronic Anterior Cruciate Ligament Lesion

**DOI:** 10.1155/2020/7436274

**Published:** 2020-03-23

**Authors:** Wen-Tzu Tang, Miao-Ju Hsu, Yi-Ming Huang, Yu-Ting Hsu, Li-Ling Chuang, Ya-Ju Chang

**Affiliations:** ^1^Graduate Institute of Athletics and Coaching Science, College of Athletics, National Taiwan Sport University, 250, Wen-Hwa 1st Rd., Kweishan, Taoyuan 33301, Taiwan; ^2^College of Exercise & Health Sciences, National Taiwan Sport University, 250, Wen-Hwa 1st Rd., Kweishan, Taoyuan 33301, Taiwan; ^3^Department of Physical Therapy, College of Health Science, Kaohsiung Medical University, 100, Shih-Chuan 1st Rd., Kaohsiung 80708, Taiwan; ^4^Department of Medical Research and Department of Physical Medicine and Rehabilitation, Kaohsiung Medical University Hospital, 100, Tzyou 1st Rd., Kaohsiung 80756, Taiwan; ^5^School of Physical Therapy and Graduate Institute of Rehabilitation Science, College of Medicine and Healthy Aging Research Center, Chang Gung University, 259, Wen-Hwa 1st Rd., Kweishan, Taoyuan 33302, Taiwan; ^6^Department of Physical Medicine and Rehabilitation, Chang Gung Memorial Hospital, Linkou, 5, Fuhsing Street, Kweishan, Taoyuan 33305, Taiwan; ^7^Neuroscience Research Center, Chang Gung Memorial Hospital, Linkou, 5, Fuhsing Street, Kweishan, Taoyuan 33305, Taiwan

## Abstract

**Purpose:**

This study is aimed at investigating the effect of low-intensity electrical stimulation on the voluntary activation level (VA) and the cortical facilitation/inhibition of quadriceps in people with chronic anterior cruciate ligament lesion.

**Methods:**

Twenty former athletes with unilateral ACL deficiencies (ACL group) and 20 healthy subjects (healthy control group) participated in the study. The quadriceps VA level, motor-evoked potential (MEP), short-interval intracortical inhibition (SICI), and intracortical facilitation (ICF) elicited by transcranial magnetic stimulation were tested before and after 30 minutes of low-intensity electrical stimulation (ES).

**Results:**

Before ES, the quadriceps VA in the ACL lesion legs of the ACL group was lower compared to the legs of the healthy control group (*P* < 0.05). The MEP sizes in the ACL lesion legs and the healthy control were not significantly different. The ACL lesion legs showed lower SICI and higher ICF compared to the healthy control group (*P* < 0.05). After ES, the quadriceps VA level increased and the SICI-ICF was modulated only in the ACL lesion legs (*P* < 0.05) but not in the healthy controls.

**Conclusions:**

Low-intensity ES can normalize the modulation of intracortical inhibition and facilitation, thereby ameliorating the activation failure in individuals with ACL lesion.

## 1. Introduction

The anterior cruciate ligament (ACL) is vulnerable to sports injury and usually leads to severe quadriceps weakness. Weakness and atrophy persist for years even though reconstruction has been made [1–3]. Severe quadriceps weakness and atrophy restricts the functional performance of the knee joint and prevents athletes' return to sports competitions. Sixty-six percent of athletes returned to sports competition one year after surgical reconstruction [1], and only 55% of athletes returned to preinjury level post surgery [2]. The causes for severe quadriceps weakness in the chronic phase of ACL deficiency are not clear. Knowing the mechanism of severe quadriceps weakness at the chronic phase of ACL deficiency and developing a therapeutic strategy are important.

ACL does not only serve as a mechanical stabilizer but also provides essential afferent input. Animal studies suggested that the loading of ACL has an excitatory effect on the thigh muscles [4], and the mechanism is possibly related to the feedback from mechanoreceptors in the ACL to gamma motor neurons. This mechanism is suggested to be important for recruiting high-threshold motor units during voluntary quadriceps contractions [5]. After ACL injury, feedback from the mechanoreceptors in the ACL is disrupted [6], resulting in a decrease of motor unit recruitment in the quadriceps and a decrease in quadriceps strength [5]. Therefore, an ACL lesion might cause a neurophysiological dysfunction which is generally overlooked in rehabilitation.

The maximal voluntary contraction force (MVC) is the most frequently used strength quantification variable which contains both central and peripheral neuromuscular factors. There are several potential ways to quantify the neurological deficits. By comparing the twitch force elicited by electrical stimulation at rest and during MVC, the voluntary activation level (VA) of the quadriceps can be quantified [7, 8]. It was found that the VA decreased bilaterally following ACL deficiency [9–12]. Studies reported that the motor-evoked potentials (MEPs) elicited by TMS had a tendency to decrease in subjects with ACL deficiency, and the resting motor threshold was significantly reduced in the ACL-injured group [13]. However, the integrity of the cortical inhibition and facilitation circuitries and whether these circuitries responded to training are not clear.

Training the athletes with an ACL lesion to return to games is usually challenging. Since ACL plays a role in providing afferent input, modifying compensation caused by the decrease in afferent input due to ACL injury might be essential. Electrical stimulation is one of the potential methods to provide sensory stimulation. Back in 1995, Snyder-Mackler et al. found that high-intensity ES would have better effects than high-level volitional exercise on the restoration of quadriceps strength after surgery. However, whether the improvement was from pure peripheral muscle structure changes [14] or from compensatory sensory stimulation is not clear. More recent studies showed that motor cortex excitability was enhanced after repetitive peripheral electrical stimulation (ES) in healthy subjects [15–17]. The role of ES in modulating the cortex excitability in ACL retraining remains unclear. Therefore, this study is aimed at investigating the neurological dysfunction and restoration after ACL deficiency training using low-intensity ES. The purpose of this study was (1) to investigate the difference of cortical facilitation/inhibition function between individuals with and without ACL deficiency and (2) to study the effect of providing additional afferent input by ES on the cortical facilitation/inhibition functions and VA. To clarify the role of afferent input, we used a low-intensity electrical stimulation to avoid excessive muscle contractions.

## 2. Materials and Methods

Twenty individuals (5 females, 15 males, aged 24.1 ± 3.55 years old) with ACL deficiency (ACL group) and twenty individuals with no physical disabilities (5 females, 15 males, aged 22.3 ± 2.62 years) (healthy control group) participated in the study. There were no significant differences in age, gender, height, and weight between the two groups (Table 1). Eighteen subjects in the ACL group had ACL reconstruction using the semitendinosus graft, patella tendon graft, or artificial ligament graft, and two subjects did not have ACL repair. All subjects of the ACL group were physically active. This was confirmed by Tegner activity level scale 4 to 5 which included recreational sports to competitive sports for 2 times per week by definition. The averaged months from ACL injury to the testing date were 30.85 ± 24.04 months, and the averaged months after reconstruction were 27.44 ± 24 months (not including two individuals with ACL deficiency). The subjects in the healthy control group had no previous history of neuromuscular disease. Subjects in both the ACL and healthy control groups revealed no pain and no knee swelling one month before the testing day. All subjects participated with informed consent, and the testing protocols had been approved by our internal review board in accordance with the Helsinki Declaration.

**Figure 1:**
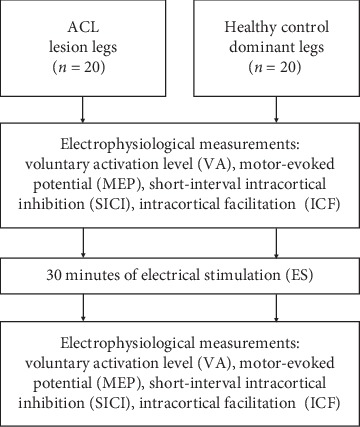
The flowchart of the study.

The ACL lesion legs of subjects in the ACL group and the dominant legs of subjects in the healthy control group were tested (Figure 1). We did not compare both legs of the healthy group since our subjects in the healthy control group did not show evidence of asymmetry on both legs. Before tests, the knee stability test was performed using a KT-2000 arthrometer and the thigh circumference was measured 10 cm above the knee joint line. Participants sat in a custom-designed chair with their hip joint fixed at 60° flexion and their knee joint fixed at 60° flexion. A force transducer maximum load of 100 kg (BA-100M, Transcell Technology Inc., IL, USA) was mounted on the custom-designed chair to measure the knee extension force. The transducer has been calibrated with the hysteresis and linearity error less than 1%. The signal from the force transducer was amplified by a transducer amplifier (Gould Inc., Valley View, OH, USA) with a gain range from 10 to 500 and a frequency response from dc to 1,000 Hz.

Surface electromyography (EMG) of the vastus medialis obliques (VMO), vastus lateralis (VL), and rectus femoris (RF) was recorded by a bipolar surface electrode with a fixed interelectrode distance of 2 cm (B&L Engineering, USA). A reference electrode was placed on the patella bone. The EMG activity was on-site preamplified with a factor of 330 and was further amplified at the mainframe. The mainframe amplifier had an input impedance greater than 10 M*Ω*, a common mode rejection ratio of 100 dB at 60 Hz, and a gain range from 0.5 to 100,000 times (Gould Bioelectric, Gould Instrument Systems Inc., USA). Both the force and EMG activity were monitored on an oscilloscope and digitized at 4000 Hz (InstruNet Model 200 PCI Controller, USA).

The femoral nerve was stimulated by a constant current stimulator (DS7A, Digitimer Ltd., England) with an active electrode (cathode, 2 cm diameter) placed at the femoral triangle and a dispersive electrode (anode, 4.5 cm × 10 cm) placed over the low back. The stimulation pulse duration was a 500 *μ*s square wave pulse, and the stimulation intensity was supramaximal, which was 110% of the intensity that elicited maximum *M* waves, resting twitches, and interpolated twitches.

After five warm-up contractions of the quadriceps, subjects were instructed to fully contract the quadriceps muscles for 3 s to measure maximum voluntary contraction (MVC) forces. They were given both verbal encouragement and visual output of their force to motivate maximal effort.

**Figure 2:**
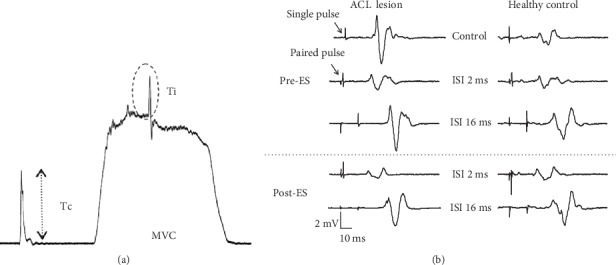
Examples of data recordings of voluntary activation level (a) and motor-evoked potentials (b) at an interstimulus interval (ISI) of 2 and 16 ms of pre- and postelectrical stimulation (ES).

For evaluating VA, the femoral nerve was stimulated at the supramaximal intensity before and during the MVC of the quadriceps to elicit unpotentiated resting twitch, interpolated twitch (Ti), and potentiated resting twitch (Figure 2(a)). The unpotentiated resting twitch and potentiated resting twitch were then averaged to obtain the control twitch (Tc) for further calculation of VA. VA was calculated from formula (1) [7, 8, 18]. The measurements of VA were repeated three times with 5 seconds in between.

(1) $$\begin{array}{c} \text{VA}=\left(1-\frac{\text{Ti}}{\text{Tc}}\right)*100\%. \end{array}$$

The motor-evoked potentials (MEPs) of VMO, VL, and RF were elicited by TMS (Magstim 200, Magstim Co., Dyfed, UK) using a double-cone coil. The optimal scalp location for consistent production of the largest MEPs in the muscle of primary interest (VMO) at the lower intensity was marked, and this location was used for the remainder of the experiment. The resting motor threshold (rMT) was defined as the minimum TMS intensity required to elicit at least five out of 10 MEPs greater or equal to 50 *μ*V in consecutive trials [19] in the relaxed VMO [19, 20].

The testing pulse intensity was set to 120% of this threshold. For a paired pulsed protocol, the conditioning stimulation intensity was set at 80% of this threshold. The interstimulation intervals were 2 and 3 ms for measuring short-interval intracortical inhibition (SICI) and 10, 13, and 16 ms for measuring ICF [18]. The MEP was measured 6 times at intervals of 9.5 to 10.5 seconds apart and in random order. To avoid the outlier from influencing the average value, the highest value and lowest value were excluded when calculating the average. The tested MEP of the paired pulse protocol was normalized by the single pulse MEP and represented as a ratio. A ratio > 1 represents a facilitation, whereas a ratio < 1 represents an inhibition.

After the above measurements, subjects received 30 minutes of electrical stimulation (ES) with two portable stimulators (multifunctional stimulator TRIO-300; Ito Co., Tokyo, Japan) on the ACL lesion legs (ACL group) or on the dominant legs (healthy control group) to activate the VM, VL, and RF. Three pairs of surface electrodes with a size of 4 cm × 5 cm were placed along the muscle belly of VMO, VL, and RF avoiding the EMG electrodes. The stimulation frequency was 25 Hz, and the pulse duration was 200 *μ*s. The on/off time was set at 1 s/1 s. The stimulation intensity was set above the sensory threshold and 1.2 times the minimal intensity to produce muscle contraction and was independently set for each muscle. After 30 minutes of ES, the VA and the single and paired MEPs were measured again.

For the single TMS protocol, the peak-to-peak amplitude of MEP was normalized to the peak-to-peak amplitude of the maximal *M* waves. For the paired TMS protocol, the testing MEPs produced by a paired TMS were expressed as a percentage of the MEP produced by a single TMS and were further averaged for each subject at each interstimulus interval (ISI) to yield individual SICI-ICF curves for an individual subject in each condition. In the individual SICI-ICI curves, ISI 2 ms and 3 ms were in the SICI range and ISI 10 ms, 13 ms, and 16 ms were in the ICF range. Since the strongest SICI and ICF might not necessarily occur in the exact same ISI for each subject in every condition, the single SICI value and ICF for each subject in each condition were extracted from the individual SICI-ICF curve. The single SICI value (SICI_max_) and single ICF were the strongest inhibition/facilitation which were the lowest value in the SICI range and the highest value in the ICF range, respectively. These values were thus used for statistical analysis (Figure 2(b)).

Data were analyzed using SAS version 9.1. The baseline difference was analyzed using a two-sample *t*-test (ACL lesion legs and healthy control) and paired *t*-test (ACL lesion legs vs. ACL nonlesion legs). The mean ± SD was calculated for outcome variables. Two-way (group by time) repeated measures ANOVA was used to evaluate the difference between groups (ACL lesion legs, healthy control) and to determine if the dependent variables were different before and after ES in the two groups (ACL lesion legs and healthy control). If a significant interaction was detected, one-way ANOVA was then applied. Tukey's post hoc test was used for analysis whenever a significant main effect was found.

## 3. Results

A KT-2000 arthrometer revealed a significant displacement difference between the lesion and nonlesion sides (lesion side: 4.62 ± 1.92mm; nonlesion side: 3.15 ± 1.41mm, *P* < 0.05), confirming that ACL lesion legs had worse knee stability than the nonlesion leg. Thigh circumference showed no significant difference between sides in the ACL group (ACL lesion legs: 43.58 ± 4.74 cm; nonlesion side: 44.78 ± 4.83 cm, *P* < 0.05).

**Figure 3:**
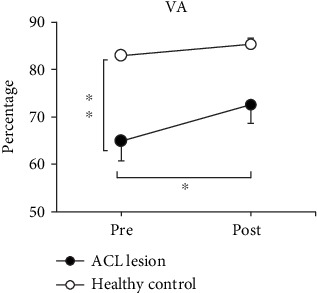
The mean ± SE of voluntary activation level (VA) before and after ES. The black circles (-●-) are ACL lesion legs, and the white circles (-○-) are legs of the healthy control group. VA is significantly increased following a 30-minute electrical stimulation (ES) only in ACL lesion legs. ^∗^Significant difference between pre- and post-ES (*P* < 0.05). ^∗∗^Significant difference between ACL lesion legs and legs of the healthy control group before ES (*P* < 0.05).

For VA (Figure 3), significant time × group interaction was shown (*F*_(1, 36)_ = 8.62, *P* = 0.006), indicating that the ACL lesion legs and the legs of the healthy control group responded differently to ES. Before ES, one-way ANOVA showed that the VA of the ACL lesion legs (64.92 ± 12.46%) was significantly lower than that of the legs of the healthy control group (*F*_(1, 36)_ = 25.12, *P* < 0.001).

After 30 minutes of ES, the VA of ACL lesion legs significantly increased from 64.92 ± 12.46% to 72.71 ± 12.47% (*F*_(1, 19)_ = 40.10, *P* < 0.001), although the value was still lower than that of the legs of the healthy control group. The ES did not alter the VA of the legs of the healthy control group (pre: 82.95 ± 9.27%, post: 85.51 ± 8.12%, *F*_(1, 17)_ = 3.97, *P* = 0.063).

**Figure 4:**
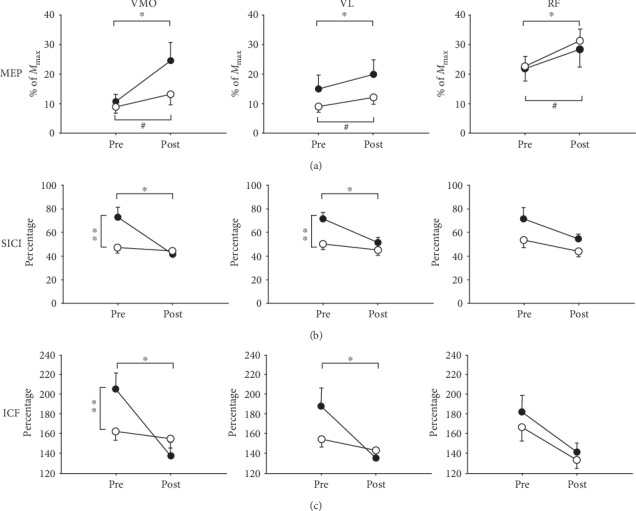
The mean ± SE of motor-evoked potential (MEP) (a), short-interval intracortical inhibition (SICI) (b), and intracortical facilitation (ICF) (c) in vastus medialis obliques (VMO), vastus lateralis (VL), and rectus femoris (RF) in the ACL lesion (-●-) and healthy control (-○-) groups before and after 30 minutes of electrical stimulation (ES) training. ^∗^Significant difference between pre- and post-ES in the ACL lesion group (*P* < 0.05). ^#^Significant difference between pre- and post-ES in the healthy control group (*P* < 0.05). ^∗∗^Significant difference between ACL lesion legs and legs of the healthy control group before ES (*P* < 0.05).

For resting MEP (Figure 4(a)), there is no significant time × group interaction in the MEP of VMO (*F*_(1, 38)_ = 3.33, *P* = 0.076), VL (*F*_(1, 38)_ = 1.95, *P* = 0.170), or RF (*F*_(1, 38)_ = 0.36, *P* = 0.552) after 30 minutes of ES, suggesting that both groups responded to ES similarly. There is no main effect of the group. These results suggested that the resting state cortical excitability was not altered in ACL lesion legs. Main effects of time showed that both ACL lesion legs and the legs of the healthy control group significantly increased MEP in VMO, VL, and RF muscles (VMO: *F*_(1, 38)_ = 11.78, *P* = 0.002; VL: *F*_(1, 38)_ = 42.78, *P* < 0.001; and RF: *F*_(1, 38)_ = 14.27, *P* < 0.001).

For SICI_max_ (Table 2), significant time × group interactions were shown in VMO (*F*_(1, 38)_ = 17.20, *P* < 0.001) and VL (*F*_(1, 38)_ = 6.48, *P* = 0.015). Before ES, the SICI_max_ of VMO in ACL lesion legs was 72.79 ± 38.78%, which was significantly higher than that of the legs of the healthy control group (47.37 ± 19.50%, *F*_(1, 38)_ = 6.68, *P* = 0.013). For VL, the value of SICI_max_ in ACL lesion legs (71.67 ± 24.05%) was significantly higher than that in the legs of the healthy control group (50.46 ± 20.41%, *F*_(1, 38)_ = 9.04, *P* = 0.005). A higher value of SICI_max_ represents a weaker SICI. The above results suggested that the ACL lesion legs had a weaker SICI than healthy control legs.

After 30 minutes of ES, in ACL lesion legs, the SICI_max_ significantly decreased from 72.79 ± 38.78% to 41.53 ± 23.99% (*F*_(1, 19)_ = 26.71, *P* < 0.001) for VMO and decreased from 71.67 ± 24.05% to 51.49 ± 20.78% for VL (*F*(1, 19) = 17.92, *P* < 0.001). These results indicate that the SICI become stronger and approached that of the healthy control group after 30 minutes of ES. No significant changes in SICI_max_ after ES were observed in the healthy control group (Figure 4(b)).

For ICF_max_ (Table 2), interactions were shown in VMO (*F*_(1, 38)_ = 11.91, *P* < 0.001) and VL (*F*_(1, 38)_ = 4.18, *P* = 0.048). Before ES, the ICF_max_ of VMO in ACL lesion legs (205.02 ± 73.47%) was significantly higher than the legs of the healthy control group (162.04 ± 38.97%, *F*_(1, 38)_ = 5.43, *P* = 0.026). The ICF_max_ of VL did not reach statistical significance (*F*_(1, 38)_ = 2.70, *P* = 0.109) between the ACL lesion legs (187.61 ± 83.95%) and the legs of the healthy control group (154.11 ± 35.65%). A higher value of ICF_max_ represents a stronger ICF.

After 30 minutes of ES, in ACL lesion legs, ICF_max_ significantly decreased from 205.02 ± 73.47% to 137.89 ± 59.85% (*F*_(1, 19)_ = 18.68, *P* < 0.001) in VMO and from 187.61 ± 83.95% to 135.12 ± 25.74% in VL (*F*_(1, 19)_ = 7.58, *P* = 0.013). These results indicate that the ICF become weaker and approached that of the healthy control group after 30 minutes of ES. Thirty minutes of ES did not change ICF_max_ in the healthy control group (Figure 4(c)).

In RF (Table 2), no significant time × group interactions were shown in either SICI_max_ (*F*_(1, 38)_ = 0.59, *P* = 0.446) or ICF_max_ (*F*_(1, 38)_ = 0.12, *P* = 0.731). A significant main effect was shown on time (SICI: *F*_(1, 38)_ = 7.01, *P* = 0.012; ICF: *F*_(1, 38)_ = 12.3, *P* = 0.001) but not on group (SICI: *F*_(1, 38)_ = 3.07, *P* = 0.088; ICF: *F*_(1, 38)_ = 0.65, *P* = 0.426). After 30 minutes of ES, both the ACL and healthy control groups showed a significant decrease in the SICI_max_ and ICF. These results indicated that ES influenced the SICI and ICF of RF to a similar extent in the ACL and healthy control groups (Figure 4).

## 4. Discussion

The results of this study showed that the ACL lesion legs had lower VA, weaker SICI, and stronger ICF in comparison to healthy people. This study also showed that the VA, MEP, SICI, and ICF can be modulated by surface ES. After 30 minutes of ES on the quadriceps muscle of the ACL lesion legs, VA was increased with the SICI and ICF approaching normal values (the SICI become stronger and the ICF become weaker).

This study provides evidences of that in the chronic phase of the ACL lesion; a severe activation failure was shown in ACL lesion legs (VA = 64.9%), even though the surgical repair had been done and the thigh circumference was not obviously different [9–12]. The amount of activation failure found in our study is comparable to a previous study which measured VA in the chronic phase but is more severe than that measured in the acute phase of the ACL lesion [10]. In Urbach et al.'s study, the subjects received 7 months of rehabilitation and still showed activation failure, suggesting that rehabilitation in acute and subacute phases of the ACL lesion might not be enough to prevent the VA loss in the chronic phase of ACL injury. In our study, all subjects had received routine rehabilitation in the acute and subacute stages but still showed VA deficits. This result suggests that activation failure should not be overlooked while performing ACL rehabilitation in the chronic phase.

The cortical reorganization might be the major contributing factor for the decrease of VA in the chronic phase of ACL injury. Our results showed a weaker intracortical inhibition and stronger intracortical facilitation in ACL lesion subjects, confirming that there is a neurological aspect of deficit and, more specifically speaking, a brain reorganization after an ACL lesion. Our study showed that the SICI and ICF changed in the ACL lesion legs. The reorganization of SICI and ICF following an ACL lesion could be related to the decrease of afferent input from the mechanoreceptors within ACL. A sensory-evoked potential study revealed a reorganization of the somatosensory cortex in a patient with an ACL lesion [21]. Deafferentation has been shown to change SICI and ICF. In subjects who had deafferentation due to amputation, decreased SICI had been reported in lower limb [22], upper limb, and forearm amputees [23, 24] and increased SICI had been reported in proximal arm amputees [23].

Our study showed that peripheral ES could increase MEP in ACL lesion subjects. Previous studies showed that ES on the upper [25] and lower extremities [15, 26] increased the MEP to 26%-50% in healthy humans [15, 25, 26]. The source of MEP facilitation was proposed within the motor cortex [27], but the changes in subcortical neural structures might not be excluded [28]. The facilitation of MEP might relate to the NMDA receptor-related synaptic plasticity and might reflect the effect of long-term potentiation in the motor cortex [25]. The present study suggests that the ACL lesion subjects showed ES-induced cortical plasticity similar to the ACL-intact subjects with the value of increment in the ACL lesion group (50-150%) somewhat greater than that in ACL-intact subjects (30-50%). The major finding of the present study was that a 30-minute ES could improve VA and normalize the abnormal SICI and ICF in individuals with an ACL lesion.

SICI and ICF are functionally important in generating muscle voluntary contraction. Zoghi et al. suggested that the SICI circuits assist the corticospinal system in producing muscle activity [29]. They suggested that selective activation of a muscle is accompanied by a selective suppression of SICI effects on the corticospinal neurons controlling that muscle. According to our results, the pre-ES SICI and ICF in ACL lesion subjects were at a less inhibited and more facilitated status. Thus, the SICI could not be further depressed to assist muscle activation and results in a decreased VA. After ES, the SICI and ICF were downregulated to the normal value and could be modulated to assist muscle activation.

Unlike the response of VMO and VL, the SICI and ICF of RF muscle were not significantly different between the ACL lesion and healthy control groups. It is possible that RF is a two-joint muscle which is different from VMO and VL which are one-joint muscles. RF in the ACL lesion group might have fewer adaptation changes due to more afferent input than VMO and VL since RF is activated during both hip and knee movements. Further study is needed to clarify this issue.

The subjects in our study were all physically active albeit VA deficient. This result suggested that people in the chronic phase of ACL injury might require a special training program other than regular physical activities to maintain VA. One might be concerned that ES might have caused fatigue that affected the results of this study. However, this should not be a concern in our study. A relatively high intensity is required as shown by a previous study using ES to induce fatigue, such as maximal or maximal tolerable intensity [30–32]. Our intensity of ES was very low which was least likely to cause fatigue. This was supported by no decrease of *M* waves shown after ES in this study.

Two of the subjects with no surgical ACL reconstruction in the ACL lesion group showed a deficit of VA that was similar to those who had ACL reconstruction. Slightly weaker SICI and stronger ICF were observed in the two subjects who did not receive ACL reconstruction when compared to the subjects with ACL reconstruction, but no statistical analysis was performed due to the small sample number. On that matter, surgical reconstruction of ACL might help preserve the functional organization of SICI and ICF but could not prevent the reorganization which is an issue that is worth further investigation.

## 5. Conclusion

After an ACL lesion, both activation failure and cortical reorganization occurred. Providing additional afferent input by 30 minutes of peripheral ES could enhance the VA of quadriceps and normalize the SICI and ICF. Preventing cortical reorganization after an ACL lesion should not be overlooked in clinical rehabilitation. Peripheral ES providing additional afferent input to compensate for the loss of afferent input due to an ACL lesion is a potential rehabilitation program.

## Figures and Tables

**Table 1:** Characteristics of the participants (mean ± SD).

	ACL group	Healthy control group	*P*
Numbers	20	20	N/A
Gender	15 men, 5 women	15 men, 5 women	N/A
Age	24.1 ± 3.55	22.3 ± 2.62	0.192
Height (cm)	173.4 ± 6.96	168.6 ± 9.16	0.241
Weight (kg)	70.4 ± 10.91	64.9 ± 11.41	0.847
ACL deficient	2		
ACL reconstruction	18		
Semitendinosus graft	11		
Patella tendon graft	6		
Artificial ligament graft	1		
ACL injury alone	7		
ACL+complex injury	13		

**Table 2:** The mean, standard deviation, and results of ANOVA of the maximal short-interval intracortical inhibition (SICI), intracortical facilitation (ICF), and *M* wave on vastus medialis obliques (VMO), vastus lateralis (VL), and rectus femoris (RF) muscles before and after 30 minutes of electrical stimulation (ES) training in different groups. The main effect is not shown if the interaction is significant (*P* < 0.05).

	Pre-ES		Post-ES		2-way ANOVA (*P*)		
	ACL lesion	Health control	ACL lesion	Healthy control	Interaction	Main effect time	Main effect group
Maximal SICI (%)							
VMO	72.79 ± 38.78	47.37 ± 19.50	41.53 ± 23.99	44.93 ± 16.17	<0.001^∗∗^	—	—
VL	71.67 ± 24.05	50.46 ± 20.41	51.49 ± 20.78	45.11 ± 18.38	0.015^∗∗^	—	—
RF	72.15 ± 42.87	54.31 ± 29.72	55.44 ± 16.68	45.13 ± 23.07	0.446	0.012^∗^	0.088
Maximal ICF (%)							
VMO	205.02 ± 73.47	162.04 ± 38.97	137.89 ± 59.85	155.02 ± 43.80	0.001^∗∗^	—	—
VL	187.61 ± 83.95	154.11 ± 35.65	135.12 ± 25.74	142.77 ± 25.20	0.048^∗∗^	—	—
RF	181.81 ± 74.87	166.44 ± 64.56	141.41 ± 40.10	133.31 ± 39.01	0.731	0.001^∗^	0.426
*M* wave (mv)							
VMO	3.28 ± 2.34	2.40 ± 1.85	3.04 ± 2.03	2.36 ± 1.78	0.237	0.114	0.225
VL	1.54 ± 1.20	1.67 ± 1.12	1.37 ± 1.08	1.57 ± 1.09	0.545	0.010^∗^	0.642
RF	0.70 ± 0.27	0.83 ± 0.36	0.72 ± 0.30	0.82 ± 0.37	0.569	0.935	0.261

^∗^Significant main effect (*P* < 0.05). ^∗∗^Significant interaction (*P* < 0.05) between time and group.

## Data Availability

The data are available from the corresponding author upon request.
